# Plasticity in the Hippocampus, Neurogenesis and Drugs of Abuse

**DOI:** 10.3390/brainsci11030404

**Published:** 2021-03-22

**Authors:** Yosef Avchalumov, Chitra D. Mandyam

**Affiliations:** 1 VA San Diego Healthcare System, San Diego, CA 92161, USA; YAvchalumov@vapop.ucsd.edu; 2 Department of Anesthesiology, University of California San Diego, San Diego, CA 92161, USA

**Keywords:** long-term potentiation, CA1, CA3, Dentate Gyrus, NPCs

## Abstract

Synaptic plasticity in the hippocampus assists with consolidation and storage of long-lasting memories. Decades of research has provided substantial information on the cellular and molecular mechanisms underlying synaptic plasticity in the hippocampus, and this review discusses these mechanisms in brief. Addiction is a chronic relapsing disorder with loss of control over drug taking and drug seeking that is caused by long-lasting memories of drug experience. Relapse to drug use is caused by exposure to context and cues associated with the drug experience, and is a major clinical problem that contributes to the persistence of addiction. This review also briefly discusses some evidence that drugs of abuse alter plasticity in the hippocampus, and that development of novel treatment strategies that reverse or prevent drug-induced synaptic alterations in the hippocampus may reduce relapse behaviors associated with addiction.

## 1. Hippocampal Formation

Hippocampus is a brain region that is important for the formation and storage of episodic and semantic declarative memories. In addition, the hippocampus is one of the most thoroughly investigated regions in the brain for learning and memory functions. Since the 1957 report of the famous H.M. case (H.M. lost the ability to form new declarative memories after surgical removal of the hippocampus which was done to treat epilepsy), the hippocampus has been at the forefront of research into the biological bases of learning and memory [1]. Our understanding of the neurophysiological bases of hippocampal function were greatly enhanced by the finding of activity-dependent synaptic plasticity in the hippocampus [2], and the discovery of hippocampal place cells—neurons that are important for spatial position of an animal [3]. These important discoveries steered many scientists and researchers around the world to investigate and subsequently advance knowledge in our understanding of the different forms of synaptic plasticity in the hippocampal circuit, and in particular how different forms of synaptic plasticity contribute to different types of memories and learning dependent on the hippocampus.

**Figure 1:**
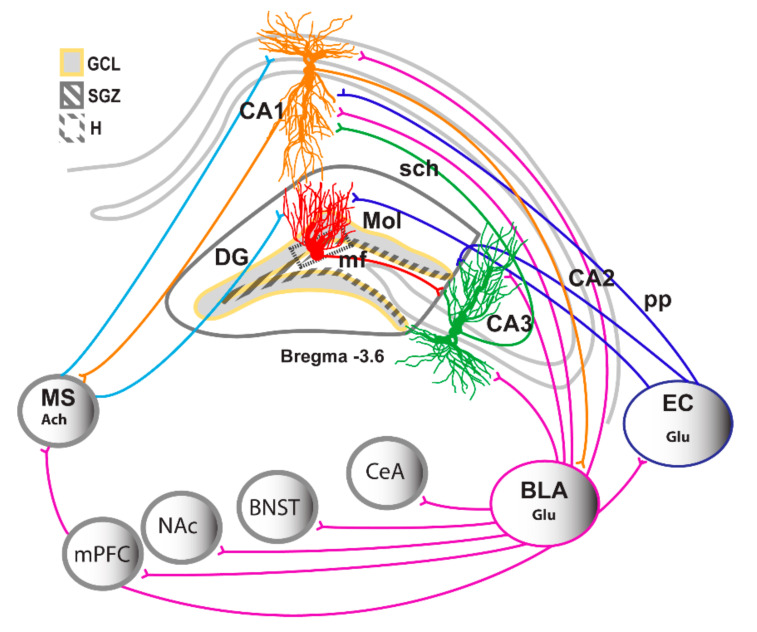
Modified from [13]. Simplified schematic representing projections in the hippocampus with emphasis on the inter- and intra- hippocampal circuits. Schematic representation of the coronal view of the hippocampus region indicating the subregions of the hippocampus and their location within the hippocampus; CA, Cornu Ammonis; CA1, CA2 and DG. Trisynaptic circuitry in the hippocampus is indicated with axons from the entorhinal cortex projecting unidirectionally to the apical dendrites of the hippocampal DG, CA1 and CA3 neurons (perforant path projection); DG neurons project to the apical dendrites of the CA3 pyramidal neurons (mossy fiber projection); CA3 neurons project to the apical dendrites of the CA1 neurons (schaffer collateral projection). The CA1 neurons have bidirectional projections to and from the BLA. The BLA also sends projections to the medial prefrontal cortex (mPFC), nucleus accumbens (NAc), bed nucleus of the stria terminalis (BNST) and central nucleus of the amygdala. The DG and CA1 also receive direct inputs from the medial septum (MS).

The hippocampus is a region in the mammalian brain that is essential for learning, including acquisition of new memories and retention of acquired memories [4,5]. It consists of three major sub-divisions: dentate gyrus (DG), cornu ammonis (CA) 1, and CA3. Information via neurotransmission is propagated from the entorhinal cortex (EC) to the DG, CA1 and CA3 (via perforant path fibers), DG to CA3 pyramidal neurons (via mossy fibers), from CA3 to CA1 pyramidal neurons (via Schaffer collaterals), and CA1 in turn projects back to the cortex in a unidirectional, feed-forward excitation manner forming the “tri-synaptic hippocampal circuit” [6,7] (Figure 1). Each sub-division plays a critical role in regulating the tri-synaptic circuit, with each neuronal subtype contributing to synaptic transmission and plasticity in the hippocampus, which contribute to the storage, consolidation, retrieval of declarative, spatial, and associative long-term memory [5,8,9,10,11]. Excitatory perforant path fibers from the EC are the major afferents projecting to the DG; therefore the DG serves as a gateway to the hippocampus by filtering and processing neurotransmission from the cortical areas [12]. In the following sections, we define plasticity in the hippocampus, and expand the information on plasticity in the CA1, CA3 and DG regions. In the subsequent sections, we introduce adult neurogenesis in the DG as a form of plasticity, and discuss how drugs of abuse effect the forms of synaptic plasticity in the hippocampus.

## 2. The What, Why and How of Plasticity in the Hippocampus

Understanding and deciphering learning and memory mechanisms is one of the major tasks for neuroscientists. To understand the cellular mechanisms of learning and memory, neuronal or synaptic plasticity in the hippocampus are studied [14]. Synaptic plasticity can be defined as the ability of neurons to change its activity in response to external or internal stimuli by reorganizing its morphology, connections or function. The term “plastic” originates from the Latin word “plasticus” which comes from the Greek word “plastos” originally meaning “formed”. In the last century several scientists made important contributions to our understanding of synaptic plasticity, including Santiago Ramon y Cajal who first defined a neuron as the anatomical, physiological, genetic unit of the nervous system in his ‘Neuron Doctrine’ [15]. Synaptic plasticity can be either strengthened or weakened. Strengthening of plasticity is commonly known as long-term potentiation (LTP), whereas weakening of plasticity is known as long-term depression (LTD, [2,16,17]). Long lasting changes in synaptic plasticity are key mechanisms underlying learning and long-term memory [18]. Another form of plasticity occurs in the brain, namely, short-term plasticity. In the context of short-term plasticity, short-term facilitation and short-term depression usually lasts from milliseconds to several minutes, and can be elicited by high or low frequency stimulations. This form of plasticity is considered to be important for short-term memory. The exact cellular and molecular mechanisms underlying short-term plasticity is not fully understood and is currently being investigated in several laboratories [19,20].

During the twentieth century, the question—how is information stored in the brain?—raised an enormous body of work that concentrated on the properties of synaptic transmission and synaptic plasticity. For example, Canadian psychologist Donald Hebb postulated a theory regarding the possible neuronal mechanisms of learning and memory [21]. Hebbian plasticity is a widely used model in the literature to study synaptic plasticity in the hippocampus in mammals. However, the first true evidence linking plasticity (short-term plasticity) to behavioral modification came from the studies in Aplysia (a marine gastropod mollusk; [18]). Research on synaptic transmission and synaptic plasticity moved beyond using invertebrates in the late 1960s and early 1970s due to discovery of novel techniques that allowed the use of mammalian brain slice preparation for electrophysiology studies [22]. This type of preparation from mammalian brain tissue was used in Andersen’s lab to discover LTP in the DG of the rabbit hippocampus [23,24]. Researchers have continued using slice preparation from hippocampal tissue in the last four decades to enhance our understanding of neuronal plasticity. The other form of synaptic plasticity, LTD was also discovered in the 1970s with use of slice preparation from mammalian brain tissue [25]. Furthermore, the development of patch clamp as well as other types of intracellular recording techniques led to discovery of different types of plasticity in the hippocampus as well as in other regions of the brain [26]. For example, during the 1990s, a new form of plasticity was discovered using intracellular recording techniques. In this form, the relative timing of active backpropagation of sodium-dependent action potential into the dendrites generated by pre- and postsynaptic neurons at monosynaptic connections was demonstrated when measured in pairs of cortical neurons [27,28]. This feature is important for a type of synaptic modification, called spike timing-dependent plasticity (STDP). STDP can be induced by timing-dependent repetitive activations of multiple spikes or even by single spike in pre and postsynaptic neurons and it was shown to occur in several brain regions [29,30,31]. STDP was also described for hippocampal synapses, and in particular, mossy fiber-CA3 and CA1 Schaffer-collaterals [32,33,34,35,36,37]. Additional forms of plasticity, such as, metaplasticity were also discovered in the hippocampus [38]. This form of plasticity is also termed as “the plasticity of synaptic plasticity” as it is a phenomenon that involves the activity-dependent changes in neuronal function that modulate synaptic plasticity. At the moment, the precise role of metaplasticity is not clear. However, several reports have indicated that it may serve to stabilize synapses within a dynamic range of activity [39,40,41,42,43,44]. Another form of plasticity was defined that regulated the total synaptic strength of a neuron and operated over longer time scales. This was termed homeostatic plasticity [45]. This type of plasticity increases or decreases the strength of all of a neuron’s synaptic inputs as a function of its activity, to maintain homeostasis over a wide range of spatial and temporal scales. This homeostatic plasticity regulates synaptic scaling and is thought to stabilize synaptic strength at the level of a single neuron [46,47]. The exact relationship between STDP and homeostatic plasticity is currently not yet understood. Additional forms of plasticity that can be visualized postmortem in the hippocampus, and several regions of the brain, is structural plasticity. Here, changes in synaptic strength causes structural modification of axons, dendrites and spine morphology. Different studies have shown that increases in spine size occurs during the induction of LTP [48], whereas spine shrinkage occurs upon LTD [49,50]. Taken together, decades of research have made clear that the mammalian brain, and particularly the hippocampus harbors several forms of plasticity, and that this ability of the hippocampus assists with hippocampus dependent function.

### 2.1. Hippocampal Circuitry

Synaptic plasticity, as discussed in the previous section, serves as a cellular mechanism for learning and storage of memory in the central nervous system. In this part of the review, we explore our current knowledge about the circuitry in the hippocampus. As indicated in the first part of the review, the hippocampal region is composed of several sub-regions, including DG, and the CA regions (CA1, CA2, and CA3). In the rat brain, there are estimated to be almost 1,000,000 DG granule neurons, 300,000 CA3 pyramidal neurons, 30,000 CA2 pyramidal neurons, and about 300,000 CA1 pyramidal neurons [7,51]. In addition to these principal excitatory neurons, there are many different types of inhibitory neurons in the hippocampus, and they constitute to about 10% to 20% of the principal neurons. We limit our discussion to the excitatory neurons in this review, and direct the readers to other publications for more information on the inhibitory neurons [52,53]. The major source of the inputs to the excitatory neurons in the hippocampus come from the EC. The EC conveys spatial and non-spatial information to the hippocampus via direct and indirect pathways [54]. Neurons in layers 2 and 3 of EC give rise to projections to the sub-regions in the hippocampus [54]. For example, layer 2 cells of EC project to DG and CA3, whereas layer 3 cells project to CA1 and the subiculum [55]. The layer 2 cells of the EC give rise to the well-characterized indirect route of information that flows through the trisynaptic pathway. In this pathway, layer 2 cells in EC send excitatory projections through the perforant path (PP) to granule cells in the DG. The DG neurons subsequently project to the CA3 via the mossy fibers. The CA3 neurons project to the CA1 pyramidal neurons via the Schaffer collaterals (SC) pathway. In addition to the trisynaptic pathway, other pathways have been discovered that assist with communications between the EC and hippocampal subregions. For example, the CA1 region of hippocampus also receives a direct glutamatergic input from EC layer 3 pyramidal neurons and this pathway is known as the temporoammonic pathway [56]. Another important indirect input to the hippocampus is through the nucleus reuniens of the thalamus, which forms a relay between the prefrontal cortex and the CA1 [57,58,59,60]. Recently additional within-hippocampal circuits have been identified, with studies demonstrating connections between the CA1 and the CA2 region of the hippocampus [61]. The CA2 region of the hippocampus is a small area which is located between the CA3 and CA1 regions [62]. Currently, the CA2 region remains largely unexplored, due to its small size and transitional location between CA3 and CA1 regions, although recent studies clearly show an important identity of the CA2 region [63]. Next, in addition to receiving inputs from the cortical and limbic regions, the CA1 region also provides a major output from the hippocampus, sending projections to several parts of the brain, including the subiculum, lateral septum via fornix, ventral striatum, amygdala and prefrontal cortex [5,64,65,66]. A small subset of pyramidal neurons from the CA1 also project to the retrosplenial cortex [65]. Taken together, these neurobiological circuits within the hippocampus and between the hippocampus and the brain indicate that the hippocampal circuitry is complex and recruits several brain regions to regulate learning and memory functions dependent on the hippocampus [67,68]. 

### 2.2. Synaptic Plasticity in CA1 Region of the Hippocampus

The key molecule that modulates plasticity in the CA1 is glutamate, by activating the glutamatergic receptors [69,70]. Support for this comes from seminal studies that have indicated causation between glutamatergic receptors and LTP, in that the selective antagonist DL-2-amino-5-phosphononvalerate (APV) of the NMDA-type glutamatergic receptor blocks the induction of LTP, but it has no effect on basal synaptic potentials following stimulation of the SC of the CA1 region of the hippocampus [70,71,72]. These initial studies were followed by additional studies that determined the mechanism underlying the activation of NMDA-type glutamatergic receptors (GluNs) to produce long-lasting enhancement of synaptic efficacy. For example, modulation of calcium entry by these receptors were thought to play a major role in regulating synaptic plasticity [73,74,75,76]. Support for this came from a report which showed that intracellular injection of the calcium chelator N, N, N’,N’-tetraacetic acid (EGTA) into pyramidal cells of the CA1 region blocks the induction of LTP in the CA1 region of the hippocampus [73]. Later studies determined the subunit of the GluN receptors that played a dominant role in regulating LTP. For example, both GluN2A and GluN2B subunits activate signaling pathways that are required for initiation and maintenance of LTP and LTD in the CA1 [77,78,79,80,81]. It is also conceptualized that differential kinetics of GluN2A and GluN2B-mediated excitatory post synaptic currents and the resulting differences in calcium influx may contribute to their roles in LTP versus LTD [82,83]. Furthermore, it is believed that different intracellular signaling pathways may contribute to the direction of synaptic changes, including LTP and LTD [84]. For example, phosphorylation of calcium/calmodulin-dependent protein kinase ll, protein kinase A (PKA), protein kinase B, extracellular signal-regulated kinases are mediators for GluN-dependent LTP [16,85,86,87,88,89,90]. However, activation of protein phosphatases, low calcium concentrations, and dephosphorylation of PKA and protein kinase B mediate GluN-dependent LTD [91,92,93,94,95,96]. Therefore, it appears that LTP and LTD are extensively studied in the CA1 region, and multiple mechanisms assist with and regulate plasticity in the CA1 region.

### 2.3. Synaptic Plasticity in the CA3 Region of the Hippocampus

Unlike the GluN dependent mechanisms in the CA1 for induction and maintenance of LTP and LTD, GluN independent mechanisms underlie induction of LTP in the CA3 in the mossy fiber synapses [97,98,99]. Mossy fiber LTP does not require postsynaptic activation and it is induced purely by presynaptic activation of intracellular calcium in presynaptic terminals [98,100]. For example, it seems that this form of LTP requires activation of presynaptic kainite receptors by endogenous glutamate [101]. Furthermore, it is becoming clear that activation of PKA and presynaptic substrates that activate PKA are probably essential in mediating this form of long-term synaptic plasticity [102,103,104]. Additionally, mechanistic studies indicate that hyperpolarization-activated cation channels provide long-lasting control of transmitter release to regulate mossy fiber LTP [105,106]. Taken together, it can be conceptualized that significant molecular heterogeneity exists between LTP in the CA1 and mossy fiber LTP in the CA3 region, and that synaptic vesicle proteins are ultimately responsible for both the short-term and long-term regulation of neurotransmitter release in mossy fiber LTP to regulate plasticity in the CA3 region.

### 2.4. Synaptic Plasticity in the Dentate Gyrus of the Hippocampus

The DG is anatomically and functionally well characterized region of the hippocampal formation. Granule cells neurons (GCNs), the principal neurons of DG, receive their primary excitatory input from stellate cells in the EC, whose axons form the perforant pathway [107]. All EC projections to DG make glutamatergic synapses onto GCNs but with different functional properties depending on the afferents coming from the lateral perforant path (LPP) or medial perforant path (MPP) [108]. LTP in the DG is very similar to the LTP in the CA1 region of the hippocampus, given that they are both GluN dependent [2,14,109]. In addition to glutamate, several other molecules have been implicated in modulating LTP in the DG. For example, neuroligins (NL) are transmembrane cell adhesion proteins that are involved in the regulation of synaptic plasticity in the DG [110]. Mechanistic studies show that hippocampal knockdown of NL1 decreases perforant path-granule cell LTP in acute slices of the adult rat DG [111]. Furthermore, global knockdown of NL1 reduces excitatory transmission and diminishes LTP in the DG [112]. Other molecules, such as brain specific guanine nucleotide exchange factor collybistin (Cb), interacts with the synaptic scaffolding protein gephyrin to regulate LTP in the DG [113]. Mechanistically, it has been demonstrated that Cb knock out mice exhibit impairment in LTP in the DG, and that it is most likely mediated by altered function of GABAergic inhibitory synapses [114]. In addition to the dependency of LTP in the DG on the glutamatergic system, LTD in the DG is also dependent on activation of GluNs as well as L type calcium channels [115]. Furthermore, a role for the cannabinoid system is implicated in mediating LTD in the DG [116]. For example, activation of cannabinoid type 1 receptor at medial perforant path mediates LTD, and the activation of the cannabinoid receptor requires activation of metabotropic glutamate receptors [116]. These findings support the distinct mechanisms in the hippocampus sub-regions in regulating LTP, and emphasize the complex cellular heterogeneity in synaptic plasticity mechanisms that could differentially regulate behaviors dependent on the hippocampus.

## 3. Neurogenesis in the Adult DG—A Form of Plasticity

The DG is of particular interest as newly born GCNs are continuously generated via a process called neurogenesis [117,118,119]. While this form of plasticity in the DG is now being recognized as functionally significant, the presence of neurogenesis was discovered several decades ago. As indicated by Santiago Ramon y Cajal in 1913—the adult brain was initially thought to be static, the nerve paths fixed and completed; the adult brain not capable of regeneration [120]. This led to the most important dogma in neuroscience, in that neurogenesis was restricted to prenatal and early postnatal development, and that the adult mammalian brain was unable to facilitate neurogenesis. However, around the same time, Ezra Allen (1912) proposed that cell proliferation can occur in the adult mammalian brain [121]. It was only in the 1960s Joseph Altman provided the first autoradiographic evidence for the production of new neurons in the DG of the hippocampus in the adult mammalian brain [122]. In the recent years, based on consistent findings from several laboratories, it is confirmed that in the adult mammalian brain, there are two regions in which active neurogenesis occur: the olfactory system and the hippocampus. In the olfactory system, neural progenitor cells are born in the subventricular zone of the lateral ventricles and these cells migrate and differentiate into newborn neurons in the olfactory bulb [123]. In the hippocampus, neural progenitor cells are born in the subgranular zone of the DG, where they and migrate into the granule cell layer and differentiate and mature into functional excitatory GCNs [119]. The authors would like to direct the readers to reviews that in detail, discuss distinct steps of neuronal development during olfactory bulb adult neurogenesis [124,125], and DG neurogenesis [126,127,128] (Table 1). 

Adult Neurogenesis in the DG Influences Synaptic Plasticity in the DG

It is now established that the DG harbors a large number of newly generated GCNs in the adult mammalian brain [129,130,131]. GCNs are the principal cell types in the DG, and they project outside of this region to the CA3 region of the hippocampus via mossy fiber axons [107]. The first description of long-lasting potentiation in the hippocampus was described in the DG, in particular in the perforant path DG synapses by Bliss and Lomo [2]. As certain types of learning and memory, and in particular, associative type of learning and memory increases neurogenesis [132], and because LTP is thought to represent synaptic model of learning and memory, current research is focused on how synaptic plasticity in the DG is influenced by neurogenesis. Here, we provide a brief update on the recent studies that have evaluated the effects of adult neurogenesis on synaptic plasticity in DG. With respect to adult born GCNs (GCNs born via neurogenesis in the proliferative zone), there is an initial critical period (days to weeks postmitosis), during which the survival and integration of adult born GCNs is dependent on both GABAergic and glutamatergic input [133,134]. For example, data from DG slices from adult born GCNs and developmentally born GCNs or pre-existing GCNs from superficial layers show that high frequency stimulation induced robust LTP in both types of GCNs [135]. Interestingly, mechanistic studies with different LTP paradigms show that LTP induction occurs at a lower threshold in adult born GCNs. In addition, LTP induction in adult born GCNs is insensitive to GABAergic transmission, whereas LTP induction in pre-existing GCNs is blocked when GABAergic transmission is intact. With respect to glutamatergic influence on adult born GCNs, in particular, survival of adult born GCNs (weeks to months postmitosis) requires GluN receptor activation [136]. Notably, LTP in adult born GCNs is blocked by GluN2B subtype-specific antagonists or genetic deletion of GluN2Bs, demonstrating that GluN2Bs are essential for DG synaptic plasticity driven by adult born GCNs [137,138,139]. Consistently, ablation of neurogenesis prevents the induction of LTP evoked by medial perforant path stimulation in the DG of slices with intact GABAergic transmission, suggesting that synaptic plasticity in the DG is supported by adult born GCNs via a GluN dependent mechanism [137,140,141]. Additional mechanistic studies have indicated that stimulation of granule cell mossy fibers is sufficient to induce LTP in the DG, as well as increase the number of adult born GCNs, indicating that neurogenesis in the DG is regulated by synaptic plasticity in the hippocampus, as well as by efferent stimulation [142]. Furthermore, studies from conditional knockdown of neurogenesis show that adult born GCNs also play a role in DG LTD [141]. Notably, restoring neurogenesis provides complete rescue of LTP, at a much faster rate than LTD [141]. These results demonstrate that both LTP and LTD in the DG are influenced by adult born GCNs and that their integration into the pre-existing network effects the ability of pre-existing GCNs to express bidirectional synaptic plasticity. In addition to effecting the expression of synaptic plasticity in the DG, adult born GCNs also play a role in the gradual decay of hippocampal LTP (a feature assisting with the gradual decay of hippocampal dependency of associative memory over time, or memory clearance; [143]). In summary, there is now strong evidence to demonstrate that synaptic plasticity in the DG is regulated by neurogenesis. Together, these data indicate that adult born GCNs are hyperplastic and may as a result make unique contributions to DG circuits and hippocampal function.

## 4. Regulation of Plasticity in the Hippocampus by Drugs of Abuse

Substance use disorder is a chronic, relapsing disorder characterized by uncontrollable drug use, which is associated with recurrent thoughts and actions aimed at compulsively taking the drugs, loss of control over drug consumption and relapse to drug taking [144,145]. In the last several decades the mechanistic studies in animal models of addiction have mainly focused on the mesolimbic as well as on mesocortical dopamine pathways and their telencephalic projection targets and their role in development and maintenance of addiction [146,147,148,149]. However, recent studies on the link between rewarding properties of drugs of abuse and the drug memories (facilitated by drug context, cues) demonstrate that the conditioning mechanisms involved during drug experience facilitate the transition from initial drug use into eventual drug dependency [150,151]. More importantly, these studies support the involvement of the hippocampus in the development and maintenance of addiction (Table 1). For example, preclinical studies suggest that initial exposure to drugs and alcohol may enhance hippocampal function and, therefore, the formation of augmented drug-context associations that contribute to the development of addiction. In the context of this hypothesis, reinforcing doses of cocaine and nicotine enhances, whereas neurotoxic doses of methamphetamine reduces LTP in the CA1 region of the hippocampus [152,153,154]. These studies indicate that the synaptic alterations in the hippocampus by stimulants could facilitate the learning of drug-associated memories and eventual addiction to the drug. Whereas stimulants have shown to enhance CA1 LTP, alcohol and drugs (opiates, cannabinoids) that are central nervous system depressants show reduced CA1 LTP. For example, evidence from studies using animal models of moderate to severe alcohol use disorder demonstrate that chronic ethanol experience inhibits hippocampal CA1 LTP [155,156], and that this inhibition fails to recover after prolonged period of forced abstinence [157]. Reduced CA1 LTP is also evident in animals made dependent to morphine or heroin [158,159]; however, enhanced DG LTP is observed after chronic morphine treatment [160]. These studies clearly demonstrate that chronic abuse of opiates produces severe alteration in hippocampal LTP, and reveals the interesting differences between morphine in its effects on the differential modulation of hippocampal sub-region specific synaptic plasticity. With respect to cannabinoids, acute treatment with synthetic CB1/CB2 cannabinoid agonists reduces CA1 LTP [161]. Similar effects on CA1 LTP were observed in animals chronically treated with delta (9)-tetrahydrocannabinol (delta-9-THC), the psychoactive component of marijuana [162]. More interesting is the fact that the reduced CA1 LTP with chronic delta-9-THC was not recovered after weeks of withdrawal [162], suggesting that the effects of CB1 agonists on synaptic depression is not transient. In addition to altering synaptic plasticity in the CA1 and DG of the hippocampus, drugs of abuse and alcohol also effect adult neurogenesis in the DG. Based on several groundbreaking studies (Table 1), it is demonstrated that reinforcing doses of stimulants, alcohol, opiates and cannabinoids reduce the number of adult born GCNs [163,164,165,166,167,168]. Mechanistic studies have shown that drugs of abuse and alcohol reduce several aspects of neurogenesis, including reducing the number of actively dividing progenitor cells, reducing the number of proliferating cells maturing and differentiating into adult born GCNs. However, the exact functional implication of such impairment in adult born GCNs is largely unknown. It will be an interesting and exciting possibility for future research to link the alterations in adult born GCNs by drugs of abuse and the synaptic plasticity in the hippocampus. Such research may open up new therapeutic strategies to treat addiction. 

## 5. Conclusions

In summary, we have briefly reviewed the major players of synaptic plasticity in the hippocampus—a brain region important for learning and memory functions. In addition, we have provided very brief summary on the effects of drugs of abuse on various forms of plasticity in the hippocampus. Taken together, the studies reviewed here suggest that addiction to drugs of abuse can be conceptualized as a learning and memory disorder as there is evidence from preclinical studies for the involvement of hippocampus-dependent learning and memory as well as hippocampal plasticity in development and maintenance of addiction. 

## Figures and Tables

**Table 1:** Summarizes a list of review manuscripts on each of the topics/subtopics covered in the manuscript. The authors recommend these review articles for additional information on each subtopic indicated [126,127,164,168,169,170,171,172,173,174,175,176,177,178,179,180,181,182,183,184,185,186,187,188,189,190,191,192,193,194,195].

Topic/References	
**Mechanism Underlying Altered Plasticity by Drugs of Abuse**	
Solinas et al., 2019 [188]	Dopaminergic plasticity
Chiamulera et al., 2020 [172]	Glutamatergic plasticity
Marquez et al., 2017 [119]	Glutamatergic plasticity
Barker and Hines, 2020 [170]	GABAergic plasticity
Fernandez-Espejo and Nunez-Dominguez, 2019 [175]	Endocannabinoid system
Zlebnik and Cheer, 2016 [192]	Endocannabinoid system
Bali and Kenny, 2019 [169]	Transcriptional mechanism
**Drugs of Abuse and Plasticity in the Hippocampus**	
Abrahao et al., 2017 [195]	Alcohol
Nixon et al., 2010 [194]	Alcohol
Mandyam and Koob, 2012 [168]	Alcohol, stimulants
Stuber et al., 2010 [189]	Alcohol, stimulants
Kutlu and Gould, 2016 [180]	Alcohol, stimulants, cannabis
Robbins et al., 2008 [186]	Alcohol, stimulants, cannabis
Canales, 2010 [193]	Stimulants
Eisch and Harburg, 2006 [164]	Opiates, stimulants
Kenney and Gould, 2008 [179]	Nicotine
**Neurogenesis in the Hippocampus—Updates on Mechanism**	
Losurdo and Grilli, 2020 [182]	Extracellular vesicles and integration of new neurons
Jorgensen and Wang, 2020 [178]	Hormonal regulation of integration of new neurons
Bonafina et al., 2020 [171]	Extrinsic signals and integration of new neurons
Niklison-Chirou et al., 2020 [185]	Epigenetic, transcriprional and metabolic regulation of integration of new neurons
Lazutkin et al., 2019 [181]	Modeling of integration of new neurons
Doan et al., 2019 [174]	Glutamatergic system and integration of new neurons
Ge et al., 2008 [176]	Glutamatergic system and integration of new neurons
Hevner et al., 2006 [177]	Glutamatergic system and integration of new neurons
Nacher and McEwen, 2006 [184]	Glutamatergic system and integration of new neurons
Rubio-Casillas and Fernandez-Guasti, 2016 [187]	Glutamatergic system and integration of new neurons
Yao et al., 2016 [190]	Glutamatergic system and integration of new neurons
Yoneyama et al., 2011 [191]	Glutamatergic system and integration of new neurons
Enikolopov et al., 2015 [126]	Tools to study integration of new neurons
Goncalves et al., 2016 [129]	Formation and integration of new neurons
Denoth-Lippuner and Jessberger, 2021 [173]	Formation and integration of new neurons
